# Self-reported challenges to border screening of travelers for Ebola by district health workers in northern Ghana: An observational study

**DOI:** 10.1371/journal.pone.0245039

**Published:** 2021-01-05

**Authors:** John Koku Awoonor-Williams, Cheryl A. Moyer, Martin Nyaaba Adokiya

**Affiliations:** 1 Policy, Planning, Monitoring, and Evaluation, Ghana Health Service, Accra, Ghana; 2 Departmetns of Learning Health Sciences and OB/GYN, University of Michigan, Ann Arbor, Michigan, United States of America; 3 Department of Global and International Health, School of Public Health, University for Development Studies, Tamale, Ghana; The Chinese University of Hong Kong, HONG KONG

## Abstract

**Background:**

The 2013–2016 Ebola Virus Disease (EVD) outbreak remains the largest on record, resulting in the highest mortality and widest geographic spread experienced in Africa. Ghana, like many other African nations, began screening travelers at all entry points into the country to enhance disease surveillance and response. This study aimed to assess the challenges of screening travelers for EVD at border entry in northern Ghana.

**Design and methods:**

This was an observational study using epidemiological weekly reports (Oct 2014-Mar 2015) of travelers entering Ghana in the Upper East Region (UER) and qualitative interviews with 12 key informants (7 port health officers and 5 district directors of health) in the UER. We recorded the number of travelers screened, their country of origin, and the number of suspected EVD cases from paper-based weekly epidemiological reports at the border entry. We collected qualitative data using an interview guide with a particular focus on the core and support functions (e.g. detection, reporting, feedback, etc.) of the World Health Organization’s Integrated Disease Surveillance and Response system. Quantitative data was analyzed based on travelers screened and disaggregated by the three most affected countries. We used inductive approach to analyze the qualitative data and produced themes on knowledge and challenges of EVD screening.

**Results:**

A total of 41,633 travelers were screened, and only 1 was detained as a suspected case of EVD. This potential case was eventually ruled out via blood test. All but 52 of the screened travelers were from Ghana and its contiguous neighbors, Burkina Faso and Togo. The remaining 52 were from the four countries most affected by EVD (Guinea, Liberia, Sierra Leone, and Mali). Challenges to effective border screening included: inadequate personal protective equipment and supplies, insufficient space or isolation rooms and delays at the border crossings, and too few trained staff. Respondents also cited lack of capacity to confirm cases locally, lack of cooperation by some travelers, language barriers, and multiple entry points along porous borders. Nonetheless, no potential Ebola case identified through border screening was confirmed in Ghana.

**Conclusion:**

Screening for Ebola remains sub-optimal at the entry points in northern Ghana due to several systemic and structural factors. Given the likelihood of future infectious disease outbreaks, additional attention and support are required if Ghana is to minimize the risk of travel-related spread of illness.

## Background

In Ghana, evidence of imported infectious diseases through border entry points have been reported [1]. Events of public health concern affect the human population if effective systems are not put in place to prevent, detect and respond in a timely and appropriate manner to health threats [2]. Ghana was classified to have one of the highest probabilities of detecting Ebola Virus Disease (EVD) cases in West Africa due to the number of travelers from the affected countries (traveler volume of 25,272 by air travel) [3–5] coupled with porous points of entry into the country. The EVD started in December 2013 and peaked between August 2014 and the first quarter of 2015 in West Africa. This outbreak has been described as the greatest in history in terms of morbidity, mortality, and geographical extension [3, 6, 7]. In August 2014, the World Health Organization (WHO) declared a Public Health Emergency of International Concern to mobilize the global community in its efforts to bring the epidemic under control [4, 6]. This was only the third time the agency had made such a declaration in its history [4, 8]. Ivory Coast, Guinea-Bissau, Mali, and Senegal share land borders with the affected countries (Guinea, Liberia, Sierra Leone and Nigeria) in West Africa [4]. By October 2015, 28,454 cases had been detected and more than 11,100 deaths reported, mainly from Guinea, Liberia, and Sierra Leone in West Africa [7, 9]. As a result various measures were rolled out including screening, quarantine, isolation and communication to stop or slow the disease spread [10]. Border screenings are often recommendations of the WHO during epidemic or pandemics [2, 11]. The West African Ebola control measures focused on avoidance, isolation, quarantine, and patient treatment. To inhibit Ebola transmission, social and behavioral practices such as health care provision, mobility and transportation required modification [12].

Given Ebola’s variable and potentially lengthy incubation period (2 to 21 days), during which time individuals infected with EVD may remain asymptomatic and able to travel, many countries implemented point-of-entry screening as a strategy to prevent and control the spread of the disease. Screening seeks to identify travelers with symptoms consistent with EVD at an earlier stage and to respond appropriately [2, 4]. The aim of border screening is to detect infectious persons at the entry point such. Identified individuals can then be placed in isolation or prevented from traveling and spreading of the disease. It also increases public awareness and protection from particular diseases [2, 10]. The isolation of travelers identified with suspected cases of Ebola and quarantine of their contacts is implemented to delay or prevent the entry of an infected person to a country or prevent the global spread of a disease from a source country [10, 11]. It may be conducted through health declaration cards, airline transit agency notification, or inspection of travelers and fever screening using a thermometer or infrared thermal image scanners [2].

Ghana implemented screening by the port health authority at the only international airport (Kotoka International Airport) and at other main points of entry by land (vehicle transport). Ghana’s port health unit was tasked with screening all travelers by inquiring about their health status and verifying their body temperature using a thermometer. Travelers presenting with symptoms, fever, or who indicated exposure to individuals with Ebola were assessed further and referred to established isolation centers. A regional rapid response team was to be notified immediately and mobilized to obtain a blood sample for testing. The port health unit was also responsible for isolating and providing quarantine for anyone who had direct contact with a person with Ebola, including any medical personnel who had provided medical services, or who had been trained in the affected countries. The unit was also tasked with actively monitoring and providing possible quarantine for all persons with travel history to the affected countries [4, 5]. In the Upper East Region (UER) of Ghana, multiple points of entry between Burkina Faso, Togo, and Ghana created a challenging environment for successfully implementing border screening for EVD. Moreover, the evidence available about screening implementation and its effectiveness at border crossings are limited. The exit screening measures for EVD in the most affected West African countries did not identify any cases. In addition, sensitivity of screening tests were found to be zero while specificity was also very low, and confirmed cases identified out of the total numbers of travelers in West Africa were zero or extremely low [2, 13]. In spite of the reported ineffectiveness of border screening measures, it has other benefits such as discouraging travel of ill persons, raising awareness, and educating the travelers [2]. The objective of this study was to assess not only the volume of screening conducted and the resulting number of suspected cases identified, but also to identify the challenges associated with implementing EVD screening along the border of the UER in northern Ghana.

## Methods

### Study setting

Ghana is a West African nation of 29 million people who are mostly concentrated in the southern and coastal regions. Ghana is bordered by Ivory Coast to the west, Burkina Faso to the north, Togo to the east, and the Atlantic Ocean to the south [14]. Our study was conducted in Upper East Region (UER) with 13 administrative districts, a predominantly agricultural area of approximately one million inhabitants, bordered by Burkina Faso to the north and Togo to the east [15–17]. The UER is one of the five northern regions of Ghana. The total land area is about 8,842 square kilometres (2.7% of the total land area of Ghana) and has savannah woodland. However, human activities with the ecology are leading to near semi-arid conditions. The climate is characterized by one rainy season from May/June to September/October. It also has low humidity and high temperatures. There are a number of tourist attractions in the UER such as the Paga Crocodile Pond and the Bolgatanga Museum [17]. The distance from Accra to UER is about 782 km while 822 km to Paga border (the Ghana–Burkina Faso border) and 860 km to Pusiga border (Ghana–Togo border). There is one main standard road from Accra through Paga to Burkina Faso, while the road leading from Bolgatanga to Togo is in a poor state and partly untarred. Often, it takes about 12–15 hours to travel by road from Accra to either border. The UER is far from the national capital with a higher likelihood of epidemics importation due to its borders.

Ghana’s health care system is organized in a three-tier system (district/sub-district/community, regional, and national levels) and the main implementing agency is the Ghana Health Service. The smallest unit of the health system is the Community-based Health Planning and Services (CHPS) [15, 18]. Each district is served by a hospital, health centers, private and mission clinics, and CHPS Compounds. The Regional Health Directorate is responsible for the overall health service planning, organization, monitoring, supervision, evaluation, and provision of technical support to districts/municipalities [17].

### Ebola case definition during the outbreak in West Africa

During the peak of the Ebola outbreak in West Africa, the case definition was revised to reflect the prevailing situation in Ghana and distributed to all levels of the health system in the country. *Alert Case*: any ill person with onset of fever who is not responding to treatment for common causes of fever in the area; any ill person with at least one of the following signs: bleeding, bloody diarrhea, blood in the urine; or any sudden death. *Suspected case*: any person alive or dead, suffering or having suffered from a sudden onset of high fever and having had contact with a suspected, probable, or confirmed Ebola case; or any person with sudden onset of high fever and at least three of the following symptoms: headache, anorexia (loss of appetite), lethargy, aching muscles or joints, breathing difficulties, vomiting, diarrhea, stomach aches, difficulty swallowing, and hiccups. A suspected case is also any person with inexplicable bleeding or any sudden inexplicable death [19]. *Probable case*: any deceased suspected case (where it has not been possible to collect specimens for laboratory confirmation) having an epidemiological link with a confirmed Ebola case or any suspected case evaluated by a clinician. *Confirmed case*: any suspected or probable case with a positive laboratory result, laboratory-confirmed cases must test positive by reverse transcriptase-polymerase chain reaction (RT-PCR) or by detection of IgM antibodies directed against Ebola.

### Travelers health declaration on entry in Ghana

At every entry point into Ghana, travelers disembark from their flights/vessels/vehicles and they provide a completed health declaration form. The first section of the form collects personal information, contact address in Ghana, and potential symptoms of EVD. The personal information section includes name, age, sex, nationality, country of departure, country visited on this trip, passport number, flight/vessel/vehicle, and seat number. The second section deals with the contact address, location, street name, and phone number of the traveler in Ghana. The third section includes questions such as: have you had close contact with patients or suspects suffering from Ebola/Viral Haemorrhagic Fever in the past 21 days? Have you had close contact with domestic animals or wildlife in the past 21 days? Do you have any of the under-listed signs and symptoms: a) fever, b) cough, c) headache, d) vomiting, e) bodily weakness, f) diarrhea, g) sore throat and h) bleeding from any part of the body.

The port health officer then collates the number of travelers screened and detected with fever or febrile illness each day. The information is then recorded in the weekly epidemiological/surveillance report by the port health unit and submitted to the district disease control officer. The recorded information is submitted to the district health administration using weekly reporting forms. All suspected cases are referred for further investigation and confirmation. The district's rapid response team then conduct further investigation, including obtaining specimens for confirmation at Noguchi Memorial Institute for Medical Research which is the only laboratory with the capacity for confirming suspected EVD cases in Ghana.

#### Screening and decision flowchart

At each border entry, port health officers are stationed to screen travelers. All travelers disembark, health declaration form completed, and temperature measured by the health officials. Through observation, health officials also look out for Ebola symptoms. Travelers who are suspected based on the case definition are isolated at the holding or isolation room at the border entry. The district director of health service and the rapid response team are contacted immediately by telephone. The district director then reports to the regional health directorate immediately for onward reporting to the national level of a suspected case. A laboratory officer who is a member of the team visits the isolation or holding room at the border entry to collect, document, package, store and transport specimens to the reference laboratory for confirmation. After the laboratory diagnosis, feedback is provided through the regional and district levels to the border of entry.

### Study design

This was an observational study that used quantitative and qualitative data. The quantitative data was from epidemiological weekly reports of travelers entering Ghana in the UER while qualitative data was from 12 key informant interviews (7 port health officers and 5 district directors of Ghana Health Service) in the five out of 13 districts in the UER that have designated border entry points to query them on screening of travelers at the entry points. Note that in the five districts with designated entry ports, there are a total of seven points of entry: Paga border (Kassena-Nankana west district), Mognore and Missiga borders (Bawku Municipal), Zebilla border (Bawku west district), Kulungugu and Pulmakom borders (Pusiga district) and Namoo border (Bongo district).

### Eligibility of key informants

Eligibility for participation included: a) working with port health unit of the district health system in one of the five districts with a designated entry port in the UER; b) working as director of health service in one of the five districts with a designated entry port in the UER; c) involvement in EVD screening and d) completion of written consent. In each district with a border entry, the director of district health service was asked and agreed to participate in the study (total of 5 district directors). In addition, at each border entry, the responsible port health officer was also asked and agreed to participate in the study (total of 7 port health officers).

### Data collection

Quantitative data were collected from the paper-based weekly epidemiological reports collected at each border entry point (October 2014 –March 2015) and entered into a Microsoft Excel (Redmond, WA) spreadsheet. October 2014 reflects the beginning of screening at the Paga border of the Kassena-Nankana West district before the other four districts with border crossings in the UER began screening for EVD in 2015. Data recorded included the number of individuals screened, the country of origin (beginning in 2015), and the number of suspected cases of EVD.

Qualitative data were collected from 12 key informants using face-to-face interviews. The main issues addressed in the semi-structured interview guide were: a) perceived objectives of EVD screening; and b) problems associated with screening, identifying, and recording of suspected EVD. Examples of the questions included: “What is the objective of EVD screening? What are the problems associated with EVD screening?” The fieldwork was conducted from 25^th^ May - 6^th^ June 2015.

### Data analysis

Data analysis was based on the number of travelers screened for EVD and disaggregated by the three most affected countries with EVD. Frequencies of screened travelers were produced from the weekly reports, as well as the number of suspected cases of EVD. The qualitative aspect captured the perspective of the informants on the EVD screening at border entry. we produced transcripts from each interview and these were read multiple times to identify specific themes and categories using an inductive approach [20]. These were data preparation, cleaning, a close reading of texts, creation of themes or categories accordingly. All the transcripts were read by the lead author while subsample transcripts were read by the co-authors. The repeated reading by all authors addressed issues of credibility, transferability, dependability and confirmability as well as coding consistency of the findings of the study. After the reading and discussion, a coding framework was developed and transcripts were coded by the lead author. This approach allows findings to emerge from the frequent, dominant or significant themes inherent in the data [20, 21]. The purpose of the approach adopted in the qualitative analysis include to develop a brief and summary format from extensive raw data; to identify clear links between the study objective and summary findings through transparent and justifiable standard [20, 21]. Finally, the Attride-Stirling thematic network analysis framework technique was used to break up the themes into basic themes, organizing themes, and global themes of the findings. This approach was employed because of its frequent use in health and social science-related studies [20–22].

### Ethical statement

Ethical approval for the study was obtained from the Navrongo Health Research Centre Institutional Review Board (**NHRCIRB155**), Ghana. We also obtained permission from the Regional Health Directorate of the Upper East Region to conduct the study. All participants were informed about the nature and processes involved in the study, the research objectives, and the confidentiality of the data. The participation of the subjects was completely voluntary in nature, and signature/thumbprint consent was obtained from each participant.

## Results

### Quantitative data

Tables 1 and 2 show weekly epidemiological data on screened travelers at the points of entry. A total of 41,633 travelers were screened between October 2014 and March 2015. Only one suspected case of EVD was identified through border screening. The person was considered a suspected case because of high temperature (>38°C), headache, breathing difficulties, coughing with blood, and vomiting. The suspected case was a traveler from Togo screened at the Namoo border (Bongo district) point of entry, yet serum samples were negative after further investigation at the Noguchi Memorial Institute for Medical Research in Accra, Ghana.

**Table 1:** Epidemiological weekly reports on screened travelers for EVD in Upper East Region, Ghana, 2014.

Week number in 2014	No. of travelers screened	No. detained as suspected EVD
(Paga border)		
42	36	0
43	582	0
44	902	0
45	983	0
46	883	0
47	1,088	0
48	931	0
49	1,215	0
50	1,753	0
51	1,730	0
52	802	0
TOTAL	10,905	0

**Table 2:** Epidemiological weekly reports on screened travelers for EVD in Upper East Region, Ghana, 2015.

Week number in 2015	No. of travelers screened	No. of travelers suspected as EVD	No. detained as suspected EVD	Travelers from affected countries in 2015			
(7 borders)				Guineans	Liberians	Sierra Leoneans	Malians
01	1,165	0	0	7	0	6	0
02	1,940	0	0	0	0	0	0
03	1,641	1	0	0	0	0	0
04	1,286	0	0	2	0	1	1
05	1,178	0	0	0	0	0	0
06	1,504	0	0	3	0	0	0
07	2,145	0	0	0	0	0	0
08	2,910	0	0	3	0	0	0
09	2,841	0	0	0	0	0	0
10	3,291	0	0	16	1	2	0
11	3,017	0	0	4	0	0	0
12	3,368	0	0	3	0	0	0
13	4,442	0	0	2	0	1	0
	**30,633**	**1**	**0**	**40**	**1**	**10**	**1**

Screening in the UER started at the Paga point of entry in the Kassena-Nankana West district in October 2014, resulting in 10,905 individuals screened in 2014. Data were not initially recorded on the country of origin. In 2015, 30,633 travelers were screened from the seven points of entry. Fifty-two travelers originated from one of the countries affected by EVD: 40 were Guineans, 10 were Sierra Leoneans, 1 was a Liberian, and 1 was a Malian. Their contact and personal information were reported to the national level per EVD reporting protocols.

### Qualitative data

#### Knowledge of border screening

Knowledge of the key informants on border screening was established in this study. They reported on the importance and purpose of EVD border screening and emphasized its need during epidemics. Routine activities and experiences during EVD border screening were also recounted. The border screening was fairly described as effective. All key informants interviewed had knowledge of EVD symptoms and surveillance, and all understood screening objectives. As one director described the purpose of screening:

“*(It is) to be able to control and contain the disease in the event of an outbreak*. *It also aims to prevent the disease from occurring in our district*. *The screening system is fairly good*.*”* District director #1

#### Isolation of suspected cases among travelers

Informants described a system for screening travelers, isolating suspected cases, and notifying the appropriate health officials in the event of suspected cases. This information was consistent among the key informants on the Ebola epidemic and border screening due to the limited training they received. They applied the knowledge acquired during border EVD screening. Though they knew of the requirements for effective screening, some border entry points had no holding or isolation rooms. This had the potential to affect the holding of suspected cases and risk of exposure to travelers and non-travelers. To mitigate it, alternative approaches were used to retained suspected cases such as retaining them in the vehicle they were traveling while the rest of the travelers disembarked. However, this approach is described as ineffective and created inconvenience or delays to travelers who were not suspected of EVD.

*“My first action is to isolate the suspected case and report the information to the Mognore health center for onward transmission to the Municipal health administration*.*”* Port health officer #3

*“Th(is) entry point has not (had a) suspected a case of Ebola yet*, *but if a case is suspected*, *the person/traveler will be isolated despite the lack of office space and then (we will) call the nearest health facility for support*.*”* Port health officer #6

*“The only suspected traveler of EVD was retained in the car and the other passengers disembarked for the screening*. *After the screening*, *we called the district director*, *the regional team*, *and the focal person for EVD to take action*.*”* Port health officer #2

#### Documentation and reporting of travelers information

The Ghana Health Service deployed a health declaration form at the border entry points to capture traveler's information. The key informants described the process for recording data on each individual screened and detained, and reporting the data to the centralized agency in the capital city. Similar personal and health information was collected from each traveler at border entry points based on the health declaration form with specific questions essential for critical data. It is mandatory for the port health officers to submit weekly screened data to the district health directorate every Monday during the Ebola epidemic based on the Ebola classification in the integrated disease surveillance and response (IDSR) system for Ghana. The data flow is from the border entry point to the district, regional and national levels.

*“We have a system in place*. *(We record) the number of passengers screened for the week*, *specifying sex*, *country of origin*, *and phone number of the traveler*. *The data is sent to the district health administration*, *(the) regional health administration*, *and (to the) national level through the port health coordinator*.*”* Port health officer #6

*“On a weekly basis*, *I send my screened data to the regional coordinator which includes the number of travelers coming in and going out*, *males*, *and females*. *The report also includes the number of suspected Ebola cases if any and where the travelers are coming from*.*”* Port health officer #3

Despite an established system for screening and reporting, key informants indicated they received minimal feedback once data were sent. Some reported hearing back only if their weekly reports were delayed, while others said they received feedback only when higher level supervisors visited. The minimal feedback to the peripheral health system and border entry points is considered a disincentive for quality data capture, compilation, and transmission to the next level of the health system. They concluded that the decision flowchart under Ebola screening was partly adhered to.

“*We receive weekly feedback on notifiable diseases but there is no specific feedback for the purpose of EVD*.*”* District director #2

#### Challenges of EVD screening

Overall, respondents reported several challenges to EVD screening. These included inadequate personal protective equipment and supplies, insufficient space at the border crossings, and too few trained staff. In addition, respondents cited an inability to confirm cases locally, lack of cooperation by some travelers, language barriers, and multiple entry points along a non-secured border. These challenges resulted in a sense of inadequate safety and fear among the health workers at the border crossings and frontlines during the EVD screening.

*Inadequate personal protective equipment and supplies*. Informants described a variety of supplies necessary for effective screening that were either absent or insufficient. These included basic supplies such as gloves, disinfectant, non-contact thermometers, and personal protective equipment (PPEs). These reports of inadequate supplies were consistent among all key informants. This has the potential to affect the commitment of health workers. In addition, the lack of gloves and disinfectants as well as low quality of PPEs may lead to avoidance of travelers with signs and symptoms by health workers. Occasionally, health declaration forms are stocked out and key informants are unable to capture travelers' information. These contribute to ineffectiveness of the border screening as an intervention for early detection and prevention of spread of disease.

“*We have inadequate logistics such as gloves*, *disinfectants for the work of Ebola in the district*. *The port health people complained of lack of gloves for the screening of travelers at the border”*. District director #1

“*The Ebola disease is contagious*, *and the ordinary thermometers cannot be used for screening and identification of suspected cases*.*”* District director #3

*“We do not have a computer*, *photocopier or scanning machine*, *and personal protective equipment for screening*. *These are major problems …”* Port health officer #5

All district directors and port health staff reported that they received some resources (e.g. apron, gloves, face mask, sanitizer’s thermometers, nose mask, and goggles). However, they indicated that the Personal Protective Equipment (PPEs) were of low quality and inadequate.

*“We received PPEs such as apron*, *gloves*, *face mask*, *and sanitizers*. *The items were of low quality compared to what was used in the three affected countries according to the regional team that visited those countries*.*”* District director #1

*Insufficient space and isolation rooms*. To minimize the spread of infection among travelers and non-travelers, the guidelines proposed holding or isolation rooms for suspected cases of EVD at the border entry points. However, the real situation at the border entry points is different. Informants described challenges with regard to space at the border screening locations. This included a lack of both office space to store supplies and documents as well as a lack of designated areas to maintain isolation of travelers suspected to be infected with EVD. In the event of a suspected case, the risk of exposure and potential spread by infected travelers is high. The lack of isolation rooms contributes to delays, frustrations and inconvenience at the border entry points. This raises more questions about the health system capacity and preparedness to implement border entry screening for EVD or it is an action to address the call by WHO for countries to conduct border screening.

“*There is no office for port health*, *and we have to be carrying our documents around which leads to conflict between our office and Ghana Immigration Service who work at the borders*.*”* Port health officer #2.

*“There is no isolation place for suspected cases*.*”* Port health officer #3.

*“There are no holding rooms for the cases to be held for waiting on the response from the regional team*.*”* District director #3

When a suspected case of EVD presented at one of the border crossing locations, the only option for isolation was to ask the individual to remain in the vehicle.

“*There were two returnees from Libya …and one was suspected to have the signs and symptoms of EVD …*. *The health declaration forms were used to take their health and travel history*. *(They) were not allowed at the border to disembark from the vehicle in which they were traveling*.*”* District director #2

*Porous borders*. While many informants felt that the screening system was effective, several described the challenges associated with policing a long border through a largely rural, with only a few main routes but many informal entry points. The porous borders are mainly due to the socio-cultural context of the study area. Along with the formal/informal border crossings, some families stay in Ghana while undertaking socio-economic activities in Togo, Burkina Faso, or Ivory Coast or vice versa. This peculiar nature of the families makes porous border crossings a major challenge during EVD screening.

“*The screening system is fairly good*. *However*, *this is a border district with many entering points which makes it difficult for effective surveillance and screening*.*”* District director #1

*“There are many unapproved routes and porous nature of the borders prevent port health officers from identifying all travelers to screen before they cross into the country*” Port health officer #6

*“The screening is not effective because there are porous borders which the travelers can use to cross into Ghana*. *In addition*, *the motor-riders carry people across without screening*. *Overall*, *there is no true infrastructure at the border for purposes of screening*. *We are using the Ghana Immigration Service facilities to do the screening*.*”* Port health officer #3

*Too few trained staff*, *leading to delays*. While all districts with points of entry had a trained port health staff, participants described challenges associated with not having enough trained personnel to handle the volume of travelers at each border crossing, which can lead to travelers getting irritated about delays. The combined effect of inadequacies in training, inadequate staff, a high volume of travelers and incomplete recording of travelers' information render the border screening measures ineffective for early detection and prevention of suspected cases from entering the country.

“*Currently*, *I am the only worker at the border to screen and record all the travelers*.*”* Port health officer #3.

*“The majority of travelers become angry*. *They are in a hurry to go because they think the port health officers are taking (too) much of their time*.*”* Port health officer #4

Participants described difficulties in recruiting and training staff to assist with EVD screening:

*“Health workers’ apathy is a challenge in providing education and training*. *It requires repeated appeals for staff to agree to participate in training programs*. *In addition*, *there is a general lack of public health educators and overall inadequate staff*.” District director #3

*Lack of capacity to confirm EVD cases locally*. Another challenge cited by respondents was the logistics involved in confirming a potential case of EVD since the port health officers cannot take blood samples, none of the districts has the capacity to confirm suspected cases using laboratory procedures, and all confirmations must be conducted in Accra, the nation’s capital. The low capacity of the peripheral, district and regional health system to implement border screening and confirm suspected EVD cases is a major weakness. Delays in confirmation of suspected cases from the designated laboratory has the potential to increase the risk of exposure among travelers and health workers, delay treatment initiation, and quality patient management.

“*The process is that when we see a suspected case at the border*, *I will first report to the Mognore health center*, *and then the information is passed to the Bawku Municipal Health Directorate*. *A response team follows up to verify and document the case*. *The suspected case is sent to the Bawku Hospital where (a) blood sample is taken and sent to Noguchi for analysis and confirmation*.*”* Port health officer #3

This process requires rapid coordination across many different groups, as well as long wait times for eventual results.

*Lack of cooperation by some travelers*. Key informants indicated that the cooperation of travelers was an important factor in whether they could be successful in preventing the potential spread of EVD. This included travelers using approved routes for border crossing, accurately completing the forms necessary for in-country contact tracing, and having patience at border-crossing sites. The port health officers tried to contact some of the travelers after they had entered Ghana, but found that the numbers were inaccurate or falsified. The strategy of holding suspected cases at isolation rooms or in the vehicle is a major inconvenience. As a result, travelers who are uncertain of their health status are likely not to cooperate or provide inaccurate information to avoid contact tracing while in the country. In addition, the porous borders enable individuals to cross into the country without using approved routes.

*“The most important challenges are lack of cooperation by travelers (and) use of un-approved routes for crossing the borders and inadequate staff”*. Port health officer #5

“*Some people visited the affected countries and … were screened for later tracing while in Ghana*. *Unfortunately*, *the phone numbers they gave were invalid*. *The travelers forged phone numbers on the form*. *When it was detected a couple of weeks later*, *it caused panic and fear within the health system*. *However*, *the rumors of them being suspected Ebola cases turned out to be negative*.*”* District director #1

*Language barriers*. Key informants described challenges associated with language differences between travelers, limiting their ability to effectively communicate. Translators were generally not readily available. Ghana is surrounded by three French-speaking countries–Burkina Faso, Togo, and Ivory Coast as well as countries such as Mali, Niger and Guinea plus about eighty Ghanaian languages. This affects effective communication and proper completion of the health declaration forms at the border entry points. Both Ghanian and foreign travelers experienced this as a challenge.

“*There is a general language problem*. *The Ghana side of the border speaks Kuusal while travelers from Burkina Faso speak Mossi*. *In addition*, *some foreign nationals hid their nationalities*.*”* District director 5

“…*The language barrier has been a challenge with the education of travelers on EVD at the borders*.*”* District director #2

### Satisfaction with EVD screening

The majority (5/7) of the port health officers reported that they were not satisfied with the screening of travelers at the points of entry for suspected EVD cases, given the challenges described above. The key informants declared that the EVD screening at the border entry points is unsatisfactory due to inadequate isolation rooms, logistics (supplies and PPEs), to few trained staff, lack of capacity to confirm suspected EVD cases, irregular or no feedback on suspected cases to the peripheral levels, inaccurate personal information of travelers, porous borders and language barriers. The combined effect of these limitations renders the border screening for EVD ineffective and unreliable for detection and prevention of disease spread in the country.

## Discussion

This study addresses a vital global health topic–Ebola screening in the broader context of disease surveillance and response systems strengthening in low-resource settings. We found that out of more than 41,000 individuals screened for Ebola, only one was detained as a potential case. The single suspected EVD case was not confirmed after further investigation. Such a ratio of screened-to-detained travelers may reflect a true lack of febrile, symptomatic, or potentially exposed travelers, but it may also be attributable to underqualified port health officers or lack of sensitive equipment such as non-contact thermometers. Thus, there is a need to invest in the health system preparedness and capacity to early detect, contain and prevent disease spread at the border communities.

Our findings are similar to other research focused on border screening. EVD screening development and implementation in Canada found two suspected cases that also tested negative [23]. The findings of the current study are also similar to previous research which reported that EVD screening did not identify any cases in West African countries. In addition, it reported that the sensitivity and specificity of screening test for Ebola were zero or very low [2, 4, 8]. Another study argued that EVD screening of travelers at risk of exposure may be most efficient if implemented at the points of departure from countries with community-based transmission [4]. However, this intervention comes with additional cost to and delays at departure points of the affected countries [24].

### Knowledge about EVD border screening

Overall, the study demonstrated that EVD screening is carried out at different entry points in the country. These include seaports, airports, and inland border entry [11]. The purpose and importance of EVD screening are known among frontline health workers. The knowledge of EVD includes routes of transmission and behavioral implications. The screening of travelers using a thermometer is able to detect travelers presenting with fever. However, it has the potential to miss about 20% of suspected cases [13]. Despite the knowledge of Ebola among informants, we concluded that EVD screening is only fairly effective in the study area. This is due to challenges such as inadequate personal protective equipment, insufficient space, and isolation rooms, too few trained staff and delays at border entry points, lack of cooperation, language barriers and porous borders. These pose a threat to the effectiveness of EVD screening at the border entry points. This coupled with the weak health system in the country has the potential to affect detection and prevention of disease spread. This conclusion is similar to previous studies that reported that screening interventions for Ebola and SARS were found to be ineffective at early detection and prevention of disease spread [2, 25].

### Isolation of suspected cases among travelers

Generally, the study found that isolation is being implemented at some border entry points while efforts are made to establish more centres in the country. However, the 48 hours holding period for two diagnostic tests to be conducted before national and international travelers are not implemented [11]. For effective EVD screening, each border entry point is required to have an isolation centre or treatment centre for holding suspected EVD cases among travelers. However, not all districts or border entry points had isolation centres for suspected EVD cases. This leads to inconsistencies in the implementation of the isolation approach as recommended by the World Health Organization at the different border entry points in the country. In Ghana, a previous study found that regions and districts did not have EVD treatment or holding centres, though isolation or holding centres are critical in EVD preparedness and screening [1, 11]. Another study has reported the existence and utilization of EVD isolation centres and increasing case find of EVD cases [26]. Timely isolation of suspected EVD cases is vital for early detection and prevention of disease spread. In the study setting, the existing EVD screening lacks the key building blocks for effectiveness.

### Documentation and EVD screening information

EVD screening uses the recommended health declaration form to collect information from travelers. This form is similar to the traveler public health declaration form recommended for countries by WHO [11]. The frontline health workers use the integrated disease surveillance and response (IDSR) system guidelines to compile and report the number of travelers and suspected cases weekly to the next level of the health system in the country [27]. The existence of health declaration forms for collecting information from travelers and schedule epidemiological reporting is important for data compilation, reporting and feedback. This is an important aspect of EVD screening in Ghana. This study also found that the health system is unable to effectively conduct contact tracing and monitoring due to inadequate health declaration forms. Some of the respondents use their own resources to make photocopies of forms for data capture and reporting. The inability to effectively implement the WHO guidelines coupled with the weak health system makes EVD screening at the border entry points ineffective [11].

### Challenges for EVD screening

Key informants indicated a variety of challenges to effective screening, yet no known cases of EVD were identified in Ghana. Considering that Ghana was classified as the country with the highest risk of detecting suspected EVD cases [3, 4], an effective screening system was both appropriate and timely. Yet this study illustrated profound challenges associated with screening and disease surveillance. About eight (8) major areas were derived from the findings to describe challenges during EVD screening. These include; inadequate personal protective equipment and supplies, insufficient space or isolation rooms, porous borders, too few trained staff, delays at border entry points, lack of capacity to confirm suspected EVD cases locally, lack of cooperation by some travelers, and language barriers. Findings from this study raise questions about screening effectiveness at the points of entry, potentially leading to ineffectiveness in early detection and prevention of disease spread in Ghana.

It is particularly noteworthy that some of these challenges come from outside the screening system itself: some travelers conceal their personal information or falsify their contact information. The likely reasons for which travelers conceal their personal information (e.g. telephone numbers) include avoiding contact tracing when a suspected case is confirmed, to avoid quarantine or deportation to the country of origin, and lack of appropriate travel documents (e.g. fake passport). This is against the WHO recommendation for EVD screening where probable and suspected cases are to be isolated and their travel restricted to prevent the spread of the disease [11]. A previous study also reported of mistrust as an issue that can lead to concealment of information from health authorities [28]. This has the potential to affect the ability of the health system to trace such individuals and their contacts when it becomes necessary. During future outbreaks, policies that implement stiff penalties for falsification may be warranted.

Existence of porous borders is a common phenomenon in the study setting and Africa as a whole. The existence of porous borders has been observed in Uganda [28]. The reasons for porous borders include economic, cultural, and social. Some individuals reside in one country while conducting their economic (market trading, agriculture and small businesses) activities in neighboring communities across the borders. There is also evidence of marriages across or along borders of neighboring countries. These undermine formal border entry points but rather favor the informal border entry points. It also affects effective implementation of the WHO guidelines for EVD screening. Accessing the formal border entry points result in delays, increase travel time and additional financial cost. Thus, travelers prefer to use informal border crossing points. These should be considered when epidemic control measures are being developed and implemented in rural settings. This overall affects the effectiveness of EVD screening at the border entry points for suspected cases.

This study supports earlier research that found that the health systems in the most affected countries were unable to identify suspected cases or confirm them in a timely manner [2, 8]. It was repeatedly reported by the informants that weekly screened data were reported irregularly due to inadequate reporting forms. To address this problem, some port health officers traveled to the districts to make photocopies using their own funds. This particular challenge of inadequate reporting forms has been reported in previous studies [27, 29]. This is one example in which integrated disease surveillance could improve data capture: if forms included multiple diseases it is plausible there might be a higher priority placed on ensuring sufficient numbers of forms were available. Our data suggest that feedback to port health officers on the EVD screening data they provide is rarely done, which is similar throughout much of sub-Saharan Africa [27, 30, 31]. Yet an integrated system could help prioritize feedback to the districts to ensure that district officers are both providing and acting upon timely data.

The findings also showed inadequate trained staff on EVD screening and related activities. This leads to delays at the border entry points during screening. Though, efforts are being made to train staff at the regional and district levels. This finding is similar to studies that found that many frontline health workers did not receive training on EVD in the country [1, 28]. The too few trained staff is a major contributory factor to the risk of exposure among health workers and other travelers. When health workers are overburdened with work, the tendency for them to make mistakes due to poor judgment increases. Not only will potential suspected EVD cases be missed, but travelers will be delayed resulting in poor cooperation and use of informal entry points.

### Limitations

The study has several limitations. First, as the investigation was limited to districts with border entry points in the Upper East Region, the findings are not representative of the entire health system or border screening system in Ghana. Second, key informants were asked to discuss the issues associated with EVD screening, which was a key component of their job responsibilities. Thus, it could be perceived that we were asking informants to tell us how well they were doing their jobs, which has the potential to lead to social desirability bias and downplaying of challenges. However, respondents were forthcoming with challenges and we do not believe social desirability significantly biased our findings.

Despite the limitations, this study has several noteworthy strengths. First, the paper addresses a very important aspect of EVD screening that has not been well examined in the research literature: the difference between formally recommended protocols and what actually happens in practice. While the protocols for border screening are of obvious importance, it is often the on-the-ground logistics that can ultimately determine whether they are successful. The findings from this study suggest that further work is necessary to bridge the gap between screening protocols and on-the-ground logistics. Second, this study illustrates the enormous number of travelers screened to identify a single potential case of EVD. While the lack of a confirmed case of EVD is ultimately a positive outcome of the screening process, it is important for governments and national and international health organizations to recognize the enormous resources required to patrol a national border to implement effective, comprehensive screening.

These findings have important implications. First, the need for adequate training, supplies and space is paramount if border screening is to be effective, each requiring sufficient human and financial resources dedicated to the effort. Second, screening guidelines should be tailored to meet local context dynamics and geographical needs for effective implementation and outcomes. Although, we do not know where and when the next Ebola outbreak affecting West Africa will occur, the spread of COVID-19 has provided additional lessons in managing the spread of infectious diseases. There are various in predictions and projections of between countries and continents. These findings and lessons learned are important for the overall disease surveillance strengthening in Ghana, West Africa and beyond.

## Conclusion

In conclusion, screening has become vital in disease surveillance and response due to the recent EVD outbreak in West Africa. However, screening for Ebola remains sub-optimal at the points of entry in northern Ghana due to several systemic and structural factors. Given the likelihood of future infectious disease outbreaks, additional attention and support is required if Ghana is to minimize the risk of travel-related spread of illness.

## Abbreviations

CHPS: Community-based Health Planning and Services
EVD: Ebola Virus Disease
IDSR: Integrated Disease Surveillance and Response
NHRCIRB: Navrongo Health Research Centre Institutional Review Board
RNA: Ribonucleic Acid
RT-PCR: Reverse Transcriptase–Polymerase Chain Reaction
UER: Upper East Region
USA: United States of America
WHO: World Health Organization
